# Human motion component and envelope characterization via wireless wearable sensors

**DOI:** 10.1186/s42490-020-0038-4

**Published:** 2020-02-27

**Authors:** Kaitlyn R. Ammann, Touhid Ahamed, Alice L. Sweedo, Roozbeh Ghaffari, Yonatan E. Weiner, Rebecca C. Slepian, Hongki Jo, Marvin J. Slepian

**Affiliations:** 1 Department of Medicine, University of Arizona, Tucson, AZ USA; 2 Department of Civil Engineering, University of Arizona, Tucson, AZ USA; 3 Department of Biomedical Engineering, University of Arizona, Tucson, AZ USA; 4 Department of Biomedical Engineering, Northwestern University, Evanston, IL USA; 5 Department of Robotics Engineering, Worcester Polytechnic Institute, Worcester, MA USA

**Keywords:** Accelerometers, Biomechanics, Biometrics, Biotechnology, Biosensors, Biomedical measurement, Engineering in medicine and biology, Gyroscopes, Motion analysis, Wearable sensors

## Abstract

**Background:**

The characterization of limb biomechanics has broad implications for analyzing and managing motion in aging, sports, and disease. Motion capture videography and on-body wearable sensors are powerful tools for characterizing linear and angular motions of the body, though are often cumbersome, limited in detection, and largely non-portable. Here we examine the feasibility of utilizing an advanced wearable sensor, fabricated with stretchable electronics, to characterize linear and angular movements of the human arm for clinical feedback. A wearable skin-adhesive patch with embedded accelerometer and gyroscope (BioStampRC, MC10 Inc.) was applied to the volar surface of the forearm of healthy volunteers. Arms were extended/flexed for the range of motion of three different regimes: 1) horizontal adduction/abduction 2) flexion/extension 3) vertical abduction. Data were streamed and recorded revealing the signal “pattern” of movement in three separate axes. Additional signal processing and filtering afforded the ability to visualize these motions in each plane of the body; and the 3-dimensional motion envelope of the arm.

**Results:**

Each of the three motion regimes studied had a distinct pattern – with identifiable qualitative and quantitative differences. Integration of all three movement regimes allowed construction of a “motion envelope,” defining and quantifying motion (range and shape – including the outer perimeter of the extreme of motion – i.e. the envelope) of the upper extremity. The linear and rotational motion results from multiple arm motions match measurements taken with videography and benchtop goniometer.

**Conclusions:**

A conformal, stretchable electronic motion sensor effectively captures limb motion in multiple degrees of freedom, allowing generation of characteristic signatures which may be readily recorded, stored, and analyzed. Wearable conformal skin adherent sensor patchs allow on-body, mobile, personalized determination of motion and flexibility parameters. These sensors allow motion assessment while mobile, free of a fixed laboratory environment, with utility in the field, home, or hospital. These sensors and mode of analysis hold promise for providing digital “motion biomarkers” of health and disease.

## Background

Motion is a vital element of human physical capacity, necessary for a wide range of activities. However, with injury or progression of age and disease, human mobility and motion may be compromised. Characterization of motion is essential for defining, classifying, and managing a broad range of movement and physiological disorders [1–3]. In recent years, alteration in movement has become recognized as a central component not only of specific motion disorders (i.e. Parkinson’s disease, Huntington’s disease), but also in a wide range of common and chronic diseases (i.e. heart failure, diabetes, stroke, pulmonary disease) [4, 5]. As such, motion maintenence and rehabilitation has increasingly become a core part of disease management [6–9]. A crucial factor needed to facilitate motion rehabilitation in medicine is simple and accurate characterization of holistic human motion with real-time feedback.

At present, commonly utilized mobile human motion monitoring sensors are simple activity-tracking, wrist-worn devices such as the Fitbit™ or the Apple Watch™, all of which provide information as to total body translation, i.e. the total number of steps and distance traveled. Full characterization and understanding of biomechanics and range of motion, however, requires much more detailed analyses of both regional body part movement – i.e. arm or leg; as well kinetic variables of movement – i.e. acceleration, velocity, and angular rotation [10]. Changes in these elements may be associated with injury, atrophy or disease, while controlled progress of recovery is important for proper rehabilitation [11, 12].

Present motion capture technologies able to capture multiple components of human motion are limited to systems largely deployed in laboratory environments. These typically employ multi-camera video capture systems and/or require multiple components or sensors attached to the body [13–21]. As such these powerful tools are not readily utilized outside of the lab setting due to their typical fixed nature, complexity of deployment and high expense (Additional file 1: Table S1 and Table S2). Over the past few years, a new class of materials and a new field has emerged, that of stretchable electronics and on-body wearables [22, 23]. With these materials, a wide range of sensor capabilities have been demonstrated including thin-film, conformal accelerometers and gyroscopes, as well as indicators of temperature, pressure, or material properties [24–26]. Our group has been involved in early stage work with a wide range of these systems. Here, we describe a wireless, conformal patch (BioStampRC, MC10 Inc.), containing accelerometer and gyroscope elements, able to measure six degrees of freedom of motion in a single skin-adherent, wearable sensor. We hypothesized that applying this system to human volunteers would allow detailed description of their motion, specifically defining motion of the individual and/or elements of their corpus, e.g. extremity movement. To identify the capabilities of our motion capture system, we specifically determined 1) the accuracy of angular and spatial displacement of the conformal wearable system, 2) performance compared with existing standards of motion detection, 3) the ability of the system to capture three-dimensional range of motion of the human arm, 4) ability to detect changes in motion with simulated applications and 5) utility to create a user-specific “motion envelope” of the arm.

## Results

### Description of BioStamp

**Fig. 1:**
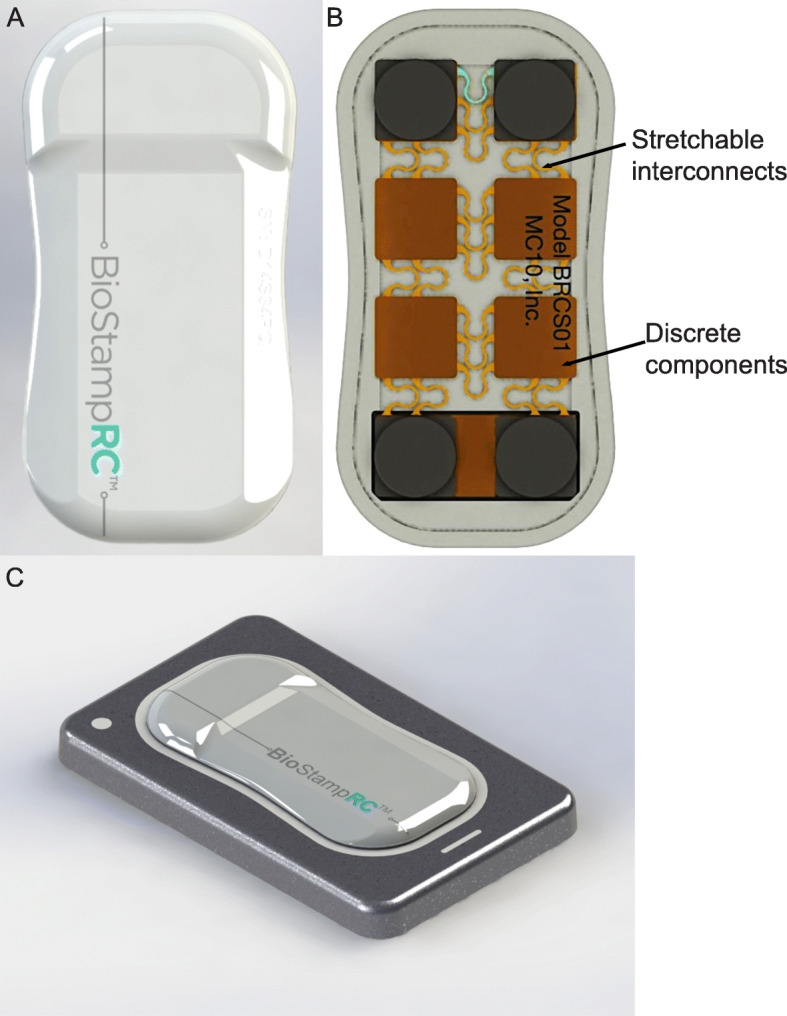
Schematic of Wearable BioStampRC. (**a**) Top view of BioStampRC (**b**) Bottom view of BioStampRC (**c**) Angled side view of BioStampRC on wireless charging platform. Images provided by MC10, Inc.

The BioStamp Research Connect (BioStampRC®; herein referred to as BioStamp) device contains flash memory (32 MB), Bluetooth Low Energy®, a low-power micro-controller unit, a rechargeable battery, and a linear and angular motion sensor for movement tracking (Fig. 1). The BioStamp was configured as a thin, pliable surface applique measuring 3.4 cm × 6.6 cm × 0.45 cm (width x length x depth). The low-power micro-controller conditions signals from the 3-axis accelerometer and gyroscope, and the sensor data are processed and sampled by the microcontroller, which transmits data into flash memory or broadcasts wirelessly via Bluetooth.

To configure and control the BioStamp device, a customized software application on a mobile device wirelessly enabled the user to set the operating parameters such as sampling rate, measurement type, and measurement range prior to data collection. The smart mobile device enabled control of data transfer from the BioStamp sensors to a cloud server for further analysis.

### Angular and spatial displacement Benchtop testing

**Fig. 2:**
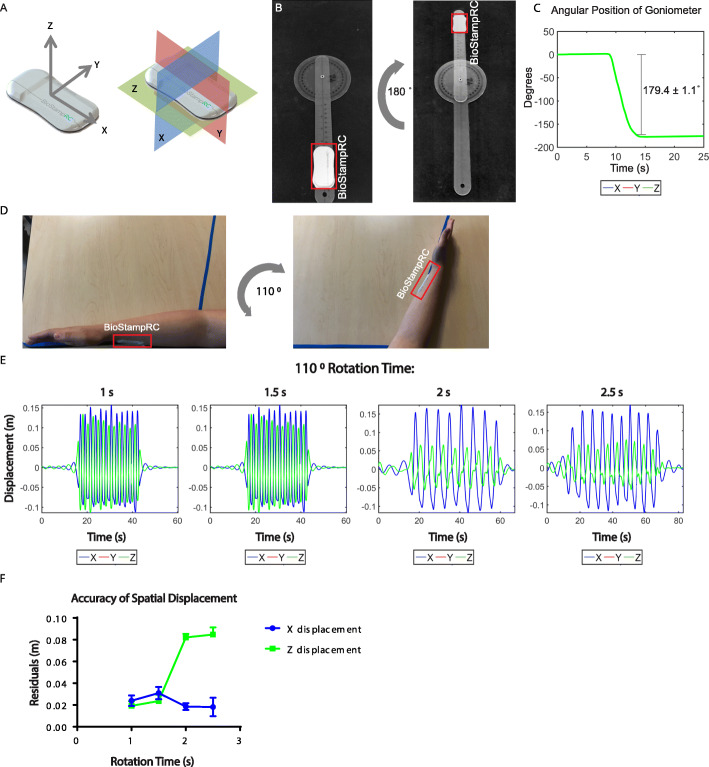
Characterization and Accuracy of BioStampRC. (**a**) Tri-axial orientation of the BioStampRC during acceleration and gyroscope recordings: x-plane (blue), y-plane (red), and zplane (green). BioStampRC image provided by MC10 Inc. (**b**) Top view of BioStampRC on distal end of goniometer on flat surface at starting position (left) and after 180 ° movement about BioStampRC z-axis. (**c**) BioStampRC angular position about z-axis after 180 ° movement on goniometer. Values shown as average degrees ± standard deviation (*n* = 3). (**d**) Top view of BioStampRC on distal volar surface of arm while on flat surface at starting position (left) and after 110 ° movement in the x-z plane, about y-axis. (**e**) Displacement output from BioStampRC accelerometer measurements after arm rotation at decreasing velocities (left to right). (**f**) Accuracy of X and Z displacement measurements at different rotational speeds. Values shown as average meters ± standard deviation (*n* ≥ 8)

Accuracy of angular displacement measured with the BioStamp was assessed by comparing to a benchtop goniometer rotating in the *z*-plane (Fig. 2a). With BioStamp adhered to the distal end of the goniometer arm, both were subjected to a 180-degree rotation as determined by the goniometer and recorded with the BioStamp (Fig. 2b). The BioStamp angular displacement measurements were obtained from integration of angular velocity acquired through the BioStamp gyroscope and were comparable (179.4 ° ± 1.1 °) to the goniometer angular displacement (*N = 3*) (Fig. 2c).

Time-dependent accuracy of spatial displacement during rotational motion was also determined with application of the BioStamp on the volar surface of a human volunteer’s forearm during 110-degree rotation about the BioStamp *y*-axis (Fig. 2d). While angular displacement was consistent during multiple (*N = 8* consecutive repetitions) rotations of the arm, error accumulation during accelerometer integration and signal processing can contribute to spatial displacement inaccuracies in the *x-* and *z-* directions (Fig. 2e). When compared to trigonometrically calculated spatial displacement of the forearm, the residuals for z- axis are higher at longer rotation times (slower angular velocity). While spatial displacement in the *z*-axis was less accurate at longer rotation times, Spatial displacement accuracy in the x-axis was unaffected by rotational speed of the arm (Fig. 2f).

### Two-dimensional limb range of motion from BioStamp

**Fig. 3:**
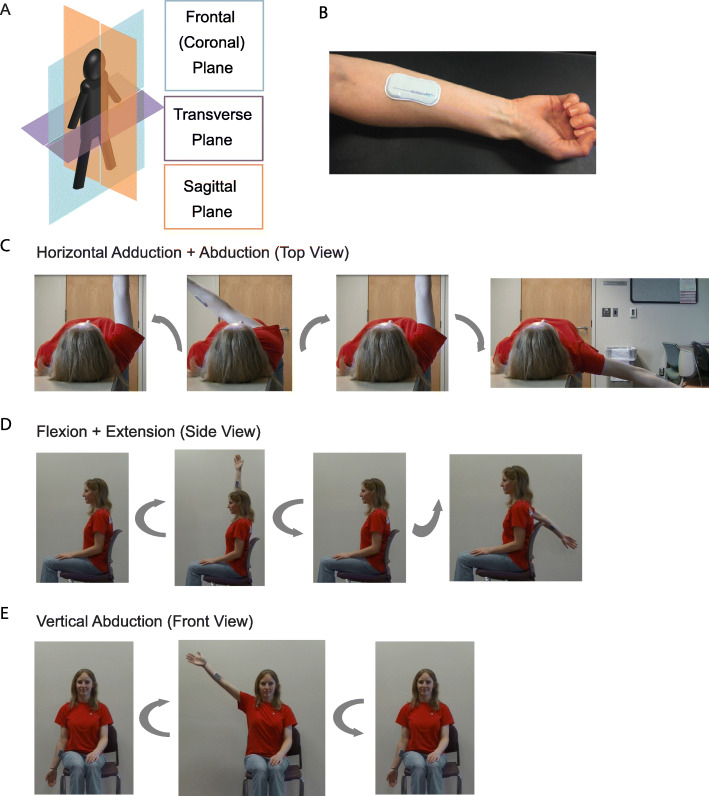
BioStampRC and Body Orientation during Motion. (**a**) Three planes of the body in anatomical position: frontal plane (blue), transverse plane (green), and sagittal plane (red). (**b**) Placement of BioStampRC on volar surface of the forearm. (**c**) Top view of horizontal adduction and abduction of arm with subject in supine position. Motion is performed with straight arm in the transverse plane and about the BioStampRC y-axis (**d**) Side view of flexion and extension of arm with subject sitting straight. Motion is performed with straight arm in the sagittal plane and about the BioStampRC z-axis. (**e**) Front view of vertical abduction of arm with subject sitting straight. Motion is performed with straight arm in the frontal plane and about the BioStampRC z-axis

The extent of motion of the arm was examined across three planes of the body: frontal, transverse, and sagittal planes (Fig. 3a). The BioStamp measured triaxial motion using both the on-board accelerometer and gyroscope. Placement of the BioStamp on the volar surface of the forearm was carefully chosen such that rotational motion of the arm would occur about a single axis of the BioStamp and within a single plane of the body.

**Fig. 4:**
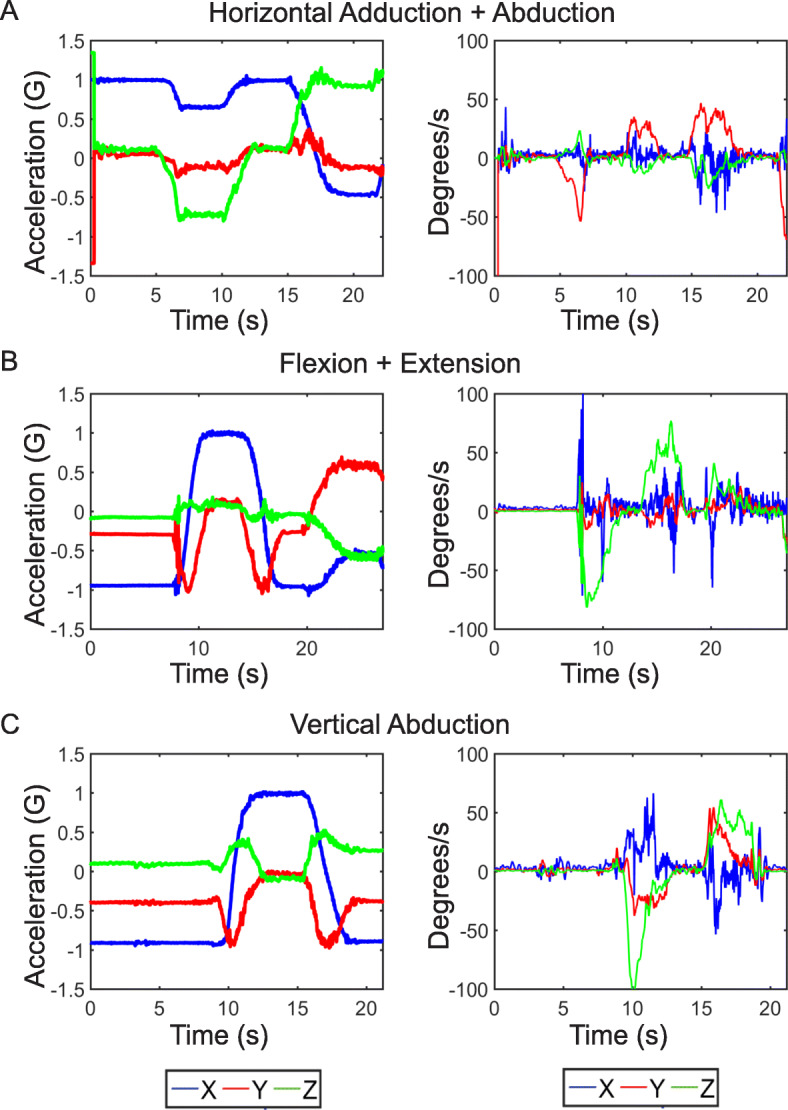
BioStampRC triaxial Motion Data. Triaxial acceleration (left) and angular velocity (right) for (**a**) horizontal abduction and adduction of the arm, (**b**) flexion and extension of the arm, and (**c**) vertical abduction of the arm

For arm range of motion in the transverse plane, horizontal adduction and abduction of the arm was performed (Fig. 3c). For arm motion in the sagittal plane of the body, flexion and extension was performed (Fig. 3d). Lastly, vertical abduction was performed to examine arm range of motion in the frontal plane (Fig. 3e). Triaxial data collected from the BioStamp during each of the planar motions exhibited distinct signatures over time (Fig. 4a-4c). For each motion, there was a single axis that exhibited a higher gyroscopic signal dependent upon the plane of rotation and the position of the subject’s arm. This axis was identified as the axis of interest for each motion type and data recorded from the corresponding BioStamp channel was used for signal integration and processing. For the horizontal motions, this was the BioStamp *y*-axis (red, Fig. 4a). For both the flexion and extension measurements and the vertical motions, this was the BioStamp *z*-axis (green, Fig. 4b and c).

**Fig. 5:**
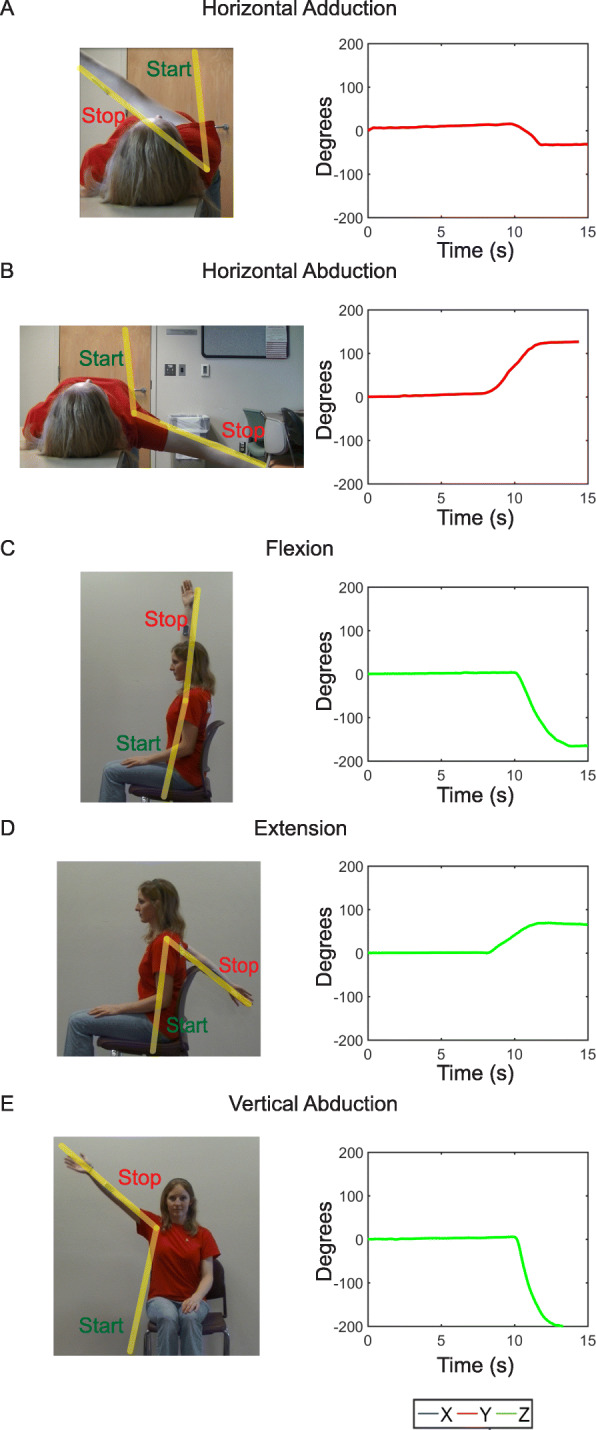
Video versus BioStampRC Data. Screenshot from motion video (left) and corresponding BioStampRC angular position (right) for (**a**) horizontal adduction of the arm about BioStampRC y-axis, (**b**) horizontal abduction of the arm about BioStampRC y-axis, (**c**) flexion of the arm about BioStampRC z-axis, (**d**) extension of the arm about BioStampRC z-axis, and (**e**) vertical abduction of the arm about BioStampRC z-axis. Yellow angles represent starting position of arm to the stopping position for each motion

Figure 5 displays the five distinct arm motions in their corresponding axes of interest for angular (gyroscopic) motion. Plots of angular positions over time show the distinct starting and stopping points of motion that could be determined from the BioStamp motion signal. Angular displacement (i.e. angular range of motion) in each plane of the body was calculated as difference between the maximum and minimum angular position for each motion. The corresponding average and deviation of the calculated ranges of motion (*N =* 3 repetitions) for each of the five motion types are shown in Table 1. Interestingly, both the largest and smallest variation in arm motion repetition were found in the transverse plane of the body; horizontal abduction had the highest variation (10.8%) and horizontal abduction had the lowest variation (3.0%). This is, in part, likely due to increased flexibility after repeated arm measurements during horizontal abduction, a motion infrequently performed by the volunteer. In contrast, variation of arm motion extent in other motion types was between 4.6 and 5.9%.

**Table 1:** Shoulder Range of Motion Measured by BioStampRC

Motion	Range of Motion from BioStampRC (Mean ± SD)
Horizontal Adduction	50.1 ± 1.5°
Horizontal Abduction	112.6 ± 12.2°
Flexion	162.8 ± 7.5°
Extension	66.7 ± 3.2°
Vertical Abduction	134.9 ± 7.9°

### Comparison of BioStamp vs. Video motion capture

The range of motion of the arm was simultaneously recorded via video camera for a visual comparison to BioStamp results. Location of the video recording was chosen such that video was taken perpendicular to the plane of motion and with the BioStamp in view (Fig. 5). Each resulting video was used to define starting and stopping point of motion, and thus corresponding angles for each motion category. While trajectory of arm motion was not the focus of this paper, representative graphs of trajectory collected from the video vs. BioStamp gyroscope are shown in Additional file 1: Figure S1.

A comparison of the measured angles for video and for BioStamp is seen in Table 2 for three separate trials. Video angular displacement measurements, all fell within two or less standard deviations of the average BioStamp measurements. Specifically, flexion, extension and vertical abduction motions were within one standard deviation of each other for most trials. Table 3 similarly displays the overall difference in angular position calculated for BioStamp and video methods in each of the three trials. The largest mean difference seen is with horizontal abduction (5.3°).

**Table 2:** BioStampRC versus Video Shoulder Range of Motion Measured in Three Separate Trials

Motion	Video Trial 1	BioStampRC Trial 1	Video Trial 2	BioStampRC Trial 2	Video Trial 3	BioStampRC Trial 3
Horizontal Adduction	50.2 ± 1.5°	48.4°	43.2 ± 4.4°	50.7°	49.1 ± 1.6°	51.2°
Horizontal Abduction	121.4 ± 2.6°	126.7°	109.6 ± 3.7°	104.8°	112.2 ± 6.3°	106.4°
Flexion	172.9 ± 4.1°	169.8°	166.3 ± 3.5°	163.7°	159.7 ± 5.9°	154.9°
Extension	69.1 ± 1.7°	69.6°	69.4 ± 1.2°	67.3°	64.3 ± 2.5°	63.2°
Vertical Abduction	128.0 ± 8.8°	130.0°	138.3 ± 11.6°	144.1°	129.3 ± 3.7°	130.7°

**Table 3:** Difference in Measured Range of Motion between BioStampRC and Video

Motion	Δangle Trial 1	Δangle Trial 2	Δangle Trial 3	Mean Δangle
Horizontal Adduction	1.8°	7.5°	2.1°	3.8 ± 3.2°
Horizontal Abduction	5.3°	4.8°	5.8°	5.3 ± 0.5°
Flexion	3.1°	2.6°	4.8°	3.5 ± 1.2°
Extension	0.5°	2.1°	1.1°	1.2 ± 0.8°
Vertical Abduction	2.0°	5.8°	1.4°	3.1 ± 2.4°

### Modeling three-dimensional range of motion – “motion envelope”

**Fig. 6:**
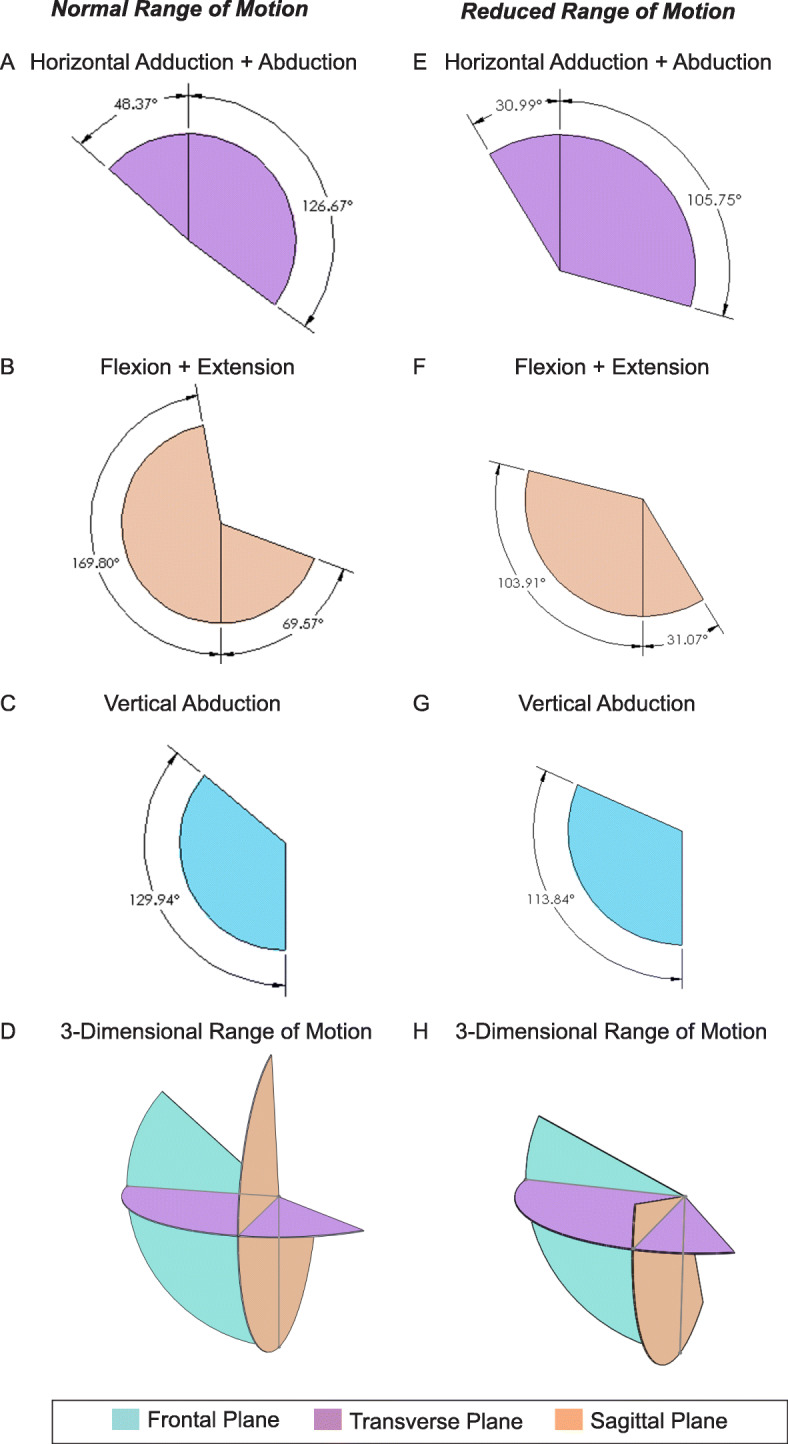
Three-Dimensional Representation of Healthy and Reduced Shoulder Range of Motion. Extent of range of movement for healthy subject in the transverse plane (**a**), sagittal plane (**b**), frontal plane (**c**) and the corresponding 3-dimensional digital representation (**d**). Extent of range of movement for subject exhibiting reduced motion in transverse plane (**e**), sagittal plane (**f**), frontal plane (**g**) and corresponding 3-dimensional digital representation (**h**)

The integrated gyroscopic values from the first BioStamp trial for each motion category were used to create a three-dimensional digital representation of the range of motion specific to the subject, i.e. a “Motion Envelope.” (Fig. 6). The largest range of motion of the arm for this subject was exhibited in the sagittal plane (Fig. 6b), followed by the transverse plane (Fig. 6a), and the frontal plane (Fig. 6c). These were combined to get a representation of the total range of motion characteristic to the subject’s shoulder joint in three axes (Fig. 6d). This process was repeated for a simulated reduced range of motion of the arm with the same volunteer (Fig. 6e-6h). Reduction in measured range of motion with the BioStamp was observed in all three planes. The frontal plane showed the largest reduction in range of motion (104.39°), followed by the transverse plane (38.30°), and frontal plane (16.10°).

**Fig. 7:**
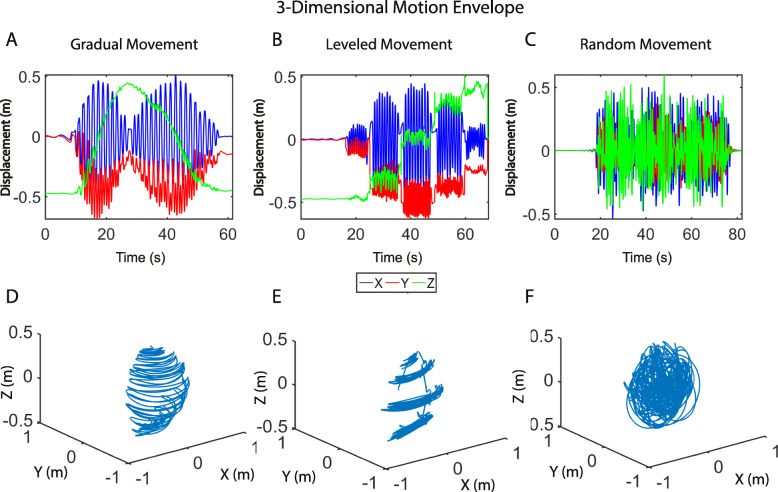
Three-Dimensional Motion Envelope of Human Shoulder. BioStampRC tri-axial arm displacement over time during gradual (**a**), leveled (**b**), and random (**c**) motion of the arm. Calculated three-dimensional displacement of arm during gradual (**d**), leveled (**e**), and random (**f**) motion of the arm

To show the comprehensive motion of the human arm, outside of the three planes of the body, three-dimensional displacement information was configured from the BioStamp accelerometer and gyroscopic data during fluid 3-dimensional arm motions. Figure 7 depicts the displacement of the arm when the user was asked to move their arm to comfortably reach the extent of their range of motion in a gradual, leveled and random manner. Whether asked to perform gradual, leveled, or random arm motion, the displacement of the arm is similar in all axes (Fig. 7a-7c). This similarity translates to comprehensive arm motion envelope in the 3-dimensional space (Fig. 7d-7f).

## Discussion

Human motion capture and quantification is crucial for detecting more granular changes in user-specific motion capacity. However, without access to non-cumbersome, simple, mobile, inexpensive systems for accurate and comprehensive feedback, the value and potential of motion evaluation is not realized, nor readily utilized as a tool for tracking valuable markers of health status. This study introduced the utility of a conformal, wireless, wearable patch system to allow capture and deconstruction of human motion into planar component elements, also facilitating the creation of a user-defined, human motion envelope. With this system, we were able to collect accurate and comprehensive motion information over time during a wide range of arm movements without the necessity of tethering to cumbersome, fixed external equipment or visualization systems.

The utilization of both accelerometers and gyroscopes during human motion capture in the tested Biostamp wearable patch system allowed for characterization of arm motion in both spatial and angular terms. However, in many motion capture studies preference for utilization of either gyroscope or accelerometer may be dependent upon the time and speed required for a motion task and the type of motion performed (i.e. planar or three-dimensional). Gyroscopes allow for simple signal processing to identify angular motion extent and velocity. However, they can experience significant signal drift over long periods of time [27, 28]. Our results suggest that the BioStamp gyroscope alone was able to capture angular displacement within one degree of accuracy when compared to a benchtop goniometer. In contrast, accelerometers provide important spatial information of motion. However, they are commonly plagued with error accumulation when integrating for spatial displacement even over small time periods and can therefore require sophisticated signal processing techniques [27–30]. The BioStamp accelerometer was able to capture spatial displacement within 2 cm. of accuracy for the limited planar motion used in this study. Despite the ability of the BioStamp accelerometer and gyroscope to independently capture accurate human arm motion, we used combined assets from both sensors in the BioStamp to allow for a comprehensive and accurate depiction of holistic human arm motion.

Apart from inertial motion sensors, visual tracking, utilizing cameras or markers placed on the human body is commonly utilized for human motion capture [31]. We chose to compare our results to visual methods by simultaneously video recording the BioStamp user perpendicular to the plane of interest, as they performed their arm motion tasks. We found, on average, the difference of our angular analysis with the BioStamp versus the visual analysis to be small (< 5.3 degrees). This is well within ranges previously explored in other visual comparison studies [32]. Similarly, all of the arm ranges captured and calculated were within normal ranges of motion for the arm previously described [33–36]. Despite this, there was clear variation in motion range between trials, as high as 22 degrees difference between trial 1 and 2 with horizontal abduction using visual methods (Table 2). Error in visual analysis enters through observer error and inability to perceive starting and ending points. Objects, such as clothing, visually obscure the joint centers and have been implicated in the variability of measurements in other studies [37]. However, the difference between trials was significantly reduced when calculating range of motion with the BioStamp, with the highest difference being 11 degrees for the same trials. While 11 degrees difference is still significant, these changes could simply be due to adjusting flexibility of the arm of the volunteer after repeated motions.

A large and inherent source of error in any type of detection of repeated motion is that of individual movement variability. This can be due either to day-to-day inconsistency in musculo-skeletal features, such as flexibility and muscle fatigue, or due to ongoing adjustment in perceptions of current and target positions [38, 39]. This perception, known as proprioception (“position sense”), is essential to motor movements [40] and includes adaptation to resistance of motion caused by three particular forces: gravity, joint structure, and the antagonist muscle and tendon systems. These aspects become more important with complex three-dimensional movements, such as the random movement for three-dimensional motion of the arm. Both the effect of gravity and the antagonist system introduce complexity into motion that causes variation during intentional human movement. Although gravity is constant, its effect on an object is dependent upon the orientation and position of that object. Thus, the effect of gravity typically changes during motion, leading to a change in the weight of the extremity and the direction and phase of the motion [41]. This issue may have been especially prevalent during horizontal abduction, due to the position of the arm and body in relation to gravity. This complexity may help explain the difficulties which a subject has in maintaining a constant range of motion within trials, but also can be more accurately accounted for using an on-board sensor, rather than indirect visual techniques. Despite high variation of range of motion quantification due to nature of the movement and proprioception, we found that the different methods of three-dimensional arm movement (gradual, leveled, or random) still produced very similar and accurate motion envelopes. Depending on the specific capability of the user and the application of the signal, any of these methods of processing with on-board sensors could be chosen as a feedback mechanism of user-specific human motion extent.

### Future directions

The scope of this study was to capture and define component motion signals of simple movements of a single limb; however, ongoing extensions of this work already demonstrate that it is possible using this system to configure a network of sensors for for whole-body capture and feedback for a series of tasks (Additional file 1: Figure S2). We hope to expand the use of the BioStamp for quantifying and defining patterns of complex motions associated with a range of activities.. Furthermore, we are continuing this work by applying these methods to other limbs or extremities (i.e. head/neck, leg/hip) in order to determine their motion envelope and elucidate further the motion extent of body segments. Use of this system in combination with feedback software system could be used to inform the subject or clinician of motion associated with disease progression or rehabilitation in comparison to user-specific “healthy” range of motion. Alternatively, with sufficient data, machine learning could be utilized to refine and establish “healthy” standards for subjects of particular demographics.

### Study limitations

As with any wearable sensor, the accuracy of the results are largely dependent upon the placement of the sensor and the ability to initiate motion from a consistent baseline. Measurements using wearable systems experience the largest errors due to inconsistent baselines, signal drift, and high noise. Where feasible, these features were corrected through signal processing. While the focus of this project was on quantifying arm range of motion, requiring only seconds to minutes of recording time, longer time periods of recording may be required for other motion capture applications. However, longer recording periods create significant error due to signal drift, rendering range of motion inaccurate. Additionally, due to the parameters of our filtering, the slower and less significant movements could result in higher errors. Post-signal processing may need to be tailored to the speed and range of the wearer’s ability in order prevent significant error accumulation.

## Conclusions

The BioStamp, a wireless, wearable motion sensor patch system, allowed for the detailed capture, analysis and definition of limb range of motion, without necessity of tethering or optical tracking. Specifically, angular and spatial displacement of the limb of the individual could be quickly and accurately assessed on a user-specific basis and integrated to create a “motion envelope.” With further translation, these limb motion envelopes can be utilized in a clinical or at-home environment for detecting changes in range of motion for quantifiable diagnostic and therapeutic assessment.

## Methods

### Device description

The BioStampRC® (Model No. BRCS01) and kit (charging station for stamps, adhesive strips, recording tablet (Samsung Galaxy Tab. A), and conductive gel), were obtained from MC10, Inc. (Lexington, MA). The BioStamp is a thin, pliable device directly applied to the skin surface (3.4 cm × 6.6 cm × 0.45 cm; weight = 7 g). The BioStamp is controlled from an embedded micro-controller unit for recording bio-signals and transmission of data via WiFi to the MC10 Investigator Portal or broadcasting wirelessly via Bluetoogh to the MC10 Discovery App, pre-loaded on the included Android™ tablet. Prior to BioStamp application to a subject, the sensor can be configured to select measurement modality (3 axis accelerometer, 3 axis gyroscope, ECG, EMG or combination), sampling frequency (50–250 Hz), and measurement range (±2–16 G for accel; ± 250–4000 °/s for gyro). Once configured, the BioStamp is applied to the subject and can be selected to start or stop recording and sync data from the tablet. Dataare then uploaded to the cloud where they can be accessed and downloaded from the MC10 Investigator Portal website. Additional specifications on the BioStamp and comparison to other wearable sensors are shown in Additional file 1: Table S1 and Table S2.

### Accuracy of BioStamp angular displacement

To show accuracy of BioStamp measurements, angular displacement was simultaneously measured using a 12-in., 360-degree goniometer. With the BioStamp adhered to the distal end of the goniometer, the goniometer was carefully rotated to a specified angle while on a flat surface. The goniometer angle was used as a reference for the calculated BioStamp angle. Angular position was determined by summation integration of the gyroscopic velocity in MATLAB (Mathworks, Inc).

### Accuracy of BioStamp spatial displacement

To show accuracy of BioStamp measurements during arm movement, spatial displacement was measured using a 12-in., 360-degree goniometer set to 110 degrees—a comfortable angle for uninhibited arm motion. With the BioStamp adhered near the wrist on the volar surface of the subject’s dominant forearm, the subject rotated their arm between the 110-degree markings for a minimum of 8 cycles at varying frequencies: 1 Hz, 0.75 Hz, 0.5 Hz, and 0.2 Hz.

### Study design

Initial studies were performed with the the Biostamp ﻿on 4 healthy volunteers (two male and two female, 22–24 years of age) to gain familiarity with signal capture and processing over a range of motions (partially previously reported [42]. Here we report an extension of this protocol examining 1) enhanced, detailed component signal analysis; and 2) reproducibility of signals for specified component (arm) motions over time. Over a three-week period a single volunteer of the initial cohort underwent follow-up analysis. All motions were repeated three times, each trial being performed a week apart. As a comparative measure, the study was also completed with the same subject exhibiting reduced range of motion. For all studies, the BioStamp was placed on the flat, volar surface of the subject’s forearm, approximately 3 in. distal from the elbow. The sensor was placed parallel to the ulnar anterior border, in the same orientation for each motion recording. To minimize error accumulation during data collection, the starting position of the arm for each motion protocol was examined from the real-time accelerometer measurements to ensure consistent orientation and position at the start of each motion study (i.e. acceleration = 1 in sensing axis feeling gravitational pull). The sensor was re-placed or the arm was adjusted if the orientation was inconsistent. Human subject approval was obtained for this study from the IRB of the University of Arizona (#1809925234).

### Arm motion protocols

#### Horizontal adduction and abduction - motion in the transverse plane

The subject began by lying in supine position on a raised surface. The subject’s dominant arm was over the edge of the raised surface such that no objects could obstruct the arm motion. Subject began with their arm straight in front of them, in the same sagittal plane as the shoulder and perpendicular to their body. Palms of the hand were facing medial to the body. This was the starting position. Recording began when subject had arm in starting position. With arm straight and palms medial, the subject adducted their arm in the transverse plane as far as possible, held for three seconds, then returned to the starting position and held until recording was paused. When subject was ready, recording resumed with arm in starting position. The subject abducted their arm horizontally in the transverse plane as far as comfortably possible, held for three seconds, and returned to the starting position until recording was completed.

#### Flexion and extension - motion in the sagittal plane

The subject began by sitting upright in a chair, facing forward with feet flat on the ground. The subject’s dominant arm was over the edge of the chair such that no objects could obstruct their arm motion. Subject began with arm straight down at their side, perpendicular to the floor. Palms of the hand were facing medial to the body. This was the starting position. Recording began when subject had arm in starting position. With arm straight and palms medial, the subject flexed their arm in the sagittal plane as far as comfortably possible, held for three seconds, and then returned to the starting position and held until recording was paused. When subject was ready, recording resumed with the arm in starting position. The subject extended their arm behind them in sagittal plane as far as comfortably possible, held for three seconds, and then returned to the starting position until recording was completed.

#### Vertical abduction - motion in the frontal plane

The subject began by sitting upright in a chair, facing forward with feet flat on the ground. The subject’s dominant arm was over the edge of the chair such that no objects could obstruct their arm motion. Subject began with arm straight down at their side, perpendicular to the floor with fifth digit of the hand medial to the body. This was the starting position. Recording began when subject had arm in starting position. With arm straight and thumbs medial, the subject vertically abducted arm in frontal plane as far as comfortably possible, held for three seconds, and then returned to the starting position and held until recording was completed.

#### Three-dimensional range of motion

The subject began standing with their arm straight down at their side. Before beginning movement, the arm was adjusted and the subject stands still for the accelerometer outputs to be as close to zero as possible. The subject was told to move their arm to reach the extent of their range of motion, comfortably. For gradual motion, the subject swung their arm laterally to medially and gradually moved their arm upwards until it was straight above their head. For leveled motion, the subject swung their arm laterally to medially approximately five times before moving it upwards and repeating the process. For random motion, the subject moved their arm to their own preference for approximately one minute.

### Three-dimensional arm spatial displacement and motion trajectory from BioStamp

3-D displacement of a body movement can be reconstructed using the acceleration and gyroscopic data from a BioStamp sensor and advanced signal processing. The BioStamp measures accelerations and gyrations in a sensor coordinate, termed as local coordinate herein, which varies with the movement of the sensor attached to a body. In such local coordinates, the acceleration contains gravity components that cause significant errors during the numerical integration process. Therefore, the integration of accelerations into displacements should require the transformation of acceleration data in a space-fixed coordinate, termed as the global coordinate here, as well as the removal of gravity components from the data. The gyroscope measures the rate of angular configuration change in the local coordinate, i.e. angular velocity ***ω*** (*ω*_*x*_, *ω*_*y*_, *ω*_*z*_) of the body, which hence can be used for coordinate transformation. It should be noted that quantities in boldface are vector quantities in here. The signal processing scheme to reconstruct 3-D global-coordinate displacement from the local-coordinate acceleration and gyroscopic measurement is as follows: the angle change *∆****θ***_*i*_ between time *t*_*i*_ and *t*_*i* + 1_ is computed as,

1 $$ \Delta  {\boldsymbol{\theta}}_i\approx \left({\boldsymbol{\omega}}_i+{\boldsymbol{\omega}}_{i+1}\right)\frac{\Delta  t}{2} $$

Euler parameters [43] *e*_0_, *e*_1_, *e*_2_, and *e*_3_ between local coordinates at time *t*_*i*_ and *t*_*i* + 1,_ are estimated as,

2 $$ {e}_0=\mathit{\cos}\left(\frac{\varnothing }{2}\right) $$

3 $$ e=\left[{e}_1,{e}_2,{e}_3\right]=\boldsymbol{n}\ \mathit{\sin}\left(\frac{\varnothing }{2}\right) $$

where ∅ = ‖***∆θ***_*i*_‖ and $\boldsymbol{n}=\frac{-\boldsymbol{\Delta }{\boldsymbol{\theta}}_i}{\varnothing }$. Then, the coordinate transformation matrix [43] for a vector quantity in the local coordinates at *t*_*i* + 1_ to *t*_*i*_ is given by,

4 $$ {\boldsymbol{A}}^i=2\left[\begin{array}{ccc}{e}_0^2+{e}_1^2-1/2& {e}_1{e}_2-{e}_0{e}_3& {e}_1{e}_3+{e}_0{e}_2\\ {}{e}_1{e}_2+{e}_0{e}_3& {e}_0^2+{e}_2^2-1/2& {e}_2{e}_3-{e}_0{e}_1\\ {}{e}_1{e}_3-{e}_0{e}_2& {e}_2{e}_3+{e}_0{e}_1& {e}_0^2+{e}_3^2-1/2\end{array}\right] $$

Thus, the acceleration 〈***a***_*i* + 1_(*a*_*x*_, *a*_*y*_, *a*_*z*_)〉^*c* = *i* + 1^, in the local coordinate at *t*_*i* + 1_, has a transformation to the local coordinate at *t*_*i*_ as,

5 $$ {\left\langle {\boldsymbol{a}}_{i+1}\right\rangle}^{c=i}={\boldsymbol{A}}^i{\left\langle {\boldsymbol{a}}_{i+1}\right\rangle}^{c=i+1} $$

Where notation 〈〉^*c* = *i*^ denotes a quantity inside the braces in the local coordinate at *t*_*i*_ .

If we assume the local coordinate at *t*_0_ (i.e. the initial coordinate) orients exactly to a fixed global coordinate, a quantity measured at the local coordinate at *t*_*i* + 1_ can be transformed in the global coordinate, or the initial coordinate at *t*_0_, as

6 $$ {\left\langle {\boldsymbol{a}}_{i+1}\right\rangle}^g={\left\langle {\boldsymbol{a}}_{i+1}\right\rangle}^{c=0}={\boldsymbol{A}}^0{\boldsymbol{A}}^1\cdots {\boldsymbol{A}}^i{\left\langle {\boldsymbol{a}}_{i+1}\right\rangle}^{c=i+1}={\mathcal{A}}^i{\left\langle {\boldsymbol{a}}_{i+1}\right\rangle}^{c=i+1} $$

Where, 〈〉^*g*^ denotes the quantity in the braces is in the global coordinate. $\kern0.50em {\mathcal{A}}^i={\boldsymbol{A}}^0{\boldsymbol{A}}^1\cdots {\boldsymbol{A}}^i$, is the transformation matrix to the global coordinate (initial coordinate at *t*_0_) from the local coordinate at *t*_*i* + 1_. Once the acceleration measurements are in the global coordinate, gravity correction is a simple operation of deducting the constant gravity components from the global acceleration data.

If we assume the body is static at the beginning (i.e. at *t*_0_), the acceleration components 〈***a***_0_(*a*_*x*_, *a*_*y*_, *a*_*z*_)〉^*c* = 0^ are solely due to the gravity. These initial acceleration components are used for gravity correction at the global coordinate.

Once the acceleration is converted in the global coordinate with the gravity correction, the displacement of the body can be reconstructed by multi-step integration and filtering process. The first integration of acceleration data results in the velocity of the body at the measured location. The resulting velocity data may still drift due to potential numerical integration errors. The drift can be removed by high-pass filtering the velocity data. Subsequent integration of the velocity data and another high-pass filtering will result in the displacement of the body motions having sufficient dynamics (i.e. 3-D random and 2-D planar motions).

For the leveled and gradual motion shown in Fig. 7D and E, further processing is required as the out-of-plane (i.e. gravitational direction) movement is too slow. Such slow out-of-plane motion components are lost due to the high pass filtering process that is necessary for drift corrections in previous steps. In this case, Euler angle, i.e. roll, and arm length (i.e. distance of the sensor from the shoulder joint) can be used to recover the out-of-plane displacement components. The roll at *t*_*i*_ can be estimated from the gravity components in the local coordinate at *t*_*i*_. The gravity components in local coordinates are estimated as,

7 $$ {\left\langle {\boldsymbol{g}}_i\right\rangle}^{c=i}={\left\langle {\boldsymbol{a}}_i\right\rangle}^{c=i}-{\left(\ {\mathcal{A}}^{i-1}\right)}^{-1}{{\left\langle {\boldsymbol{a}}_i\right\rangle}^g}_{corr} $$

where 〈***g***_*i*_〉^*i*^ is the gravity components at *t*_*i*_ in the local coordinate at *t*_*i*_, 〈***a***_*i*_〉^*g*^_*corr*_ is the acceleration after gravity correction in the global coordinate, ( )^−1^ notation denotes the matrix inverse of the quantity inside. The roll from the local gravity components at *t*_*i*_ are estimated as,

8 $$ {roll}_i= atan\left(\frac{-{\left\langle {\left({g}_x\right)}_i\right\rangle}^{c=i}}{{\left\langle {\left({g}_z\right)}_i\right\rangle}^{c=i}}\right) $$

Then the corrected *y* and *z* components of displacements are.

9 $$ {{\left\langle {y}_i\right\rangle}^g}_{corr}={\left\langle {y}_i\right\rangle}^g-l\ \mathit{\sin}\left({roll}_i\right); $$

10 $$ {{\left\langle {z}_i\right\rangle}^g}_{corr}={\left\langle {z}_i\right\rangle}^g+ lcos\left({roll}_i\right), $$

where *l* is the length of the arm.

All processing mentioned above was done in the MATLAB environment. An elliptical high-pass filter with 0.1 Hz cut-off frequency was used for this application, assuming the frequency contents of the arm motion were higher than the cut-off frequency. For other applications having different arm dynamics, the cut-off frequency can be adjusted accordingly. The schematic of the processing is summarized in Additional file 1: Figure S3.

### Arm angular displacement from BioStamp gyroscope

With BioStamp on recording from the subject’s forearm, the subject was instructed to separately perform movements of the arm in frontal, sagittal, and transverse planes. During motion performance, triaxial gyroscope and acceleration data with a sampling rate of 62.5 Hz, a gyroscopic range of − 4000°/s to + 4000°/s and acceleration range of -4G to +4G, were collected using the BioStamp. The collected gyroscopic data were integrated with respect to time for each motion in the corresponding axis of rotation to determine angular position of the arm. Total range of motion was determined by evaluating the difference in the maximum and minimum angular positions. A visual representation was created for the three motions of each plane using SolidWorks. Data collection with the BioStamp was completed and analyzed three separate times for each motion category.

### Arm angular displacement from video capture

Video was taken of the subject performing motion while wearing the BioStamp. Videos were recorded with a JVC HD Everio video camera, facing perpendicular to the axis of arm rotation. Range of motion angles were measured from video using ImageJ (NIH) with the angle tool. The angle tool measured the angles between a point on the forearm at the minimum (starting) position of the arm and the same point at the maximum (ending) position of the arm. The subject’s arm (elbow-to-wrist length) was measured and used as a standard reference point for scaling the video. Each video was analyzed three times with the angle tool, and each motion was video recorded three times. Angle measurements from a single motion video were averaged and displayed as mean ± standard deviation (*N* = 3).

## Supplementary information

**Additional file 1: Table S1.** Wearable Motion Capture Sensor Specifications. **Table S2.** Wearable Fitness Tracker Capabilities. **Figure S1.** 2-D Trajectories of Human Arm Motion. **Figure S2.** Motion Signatures of Gross Human Activity Series. Flow Diagram of Motion Data Processing.

## Abbreviations

2-D: 2-Dimensional
3-D: 3-Dimensional
HD: High-definition
MB: Megabytes
NIH: National Institute of Health
RC: Research Connect

## References

[1] WHO | International Classification of Functioning, Disability and Health (ICF). WHO [Internet]. 2017 [cited 2017 Dec 18]; Available from: http://www.who.int/classifications/icf/en/

[2] Ropper AH, Adams RD (Raymond D, Victor M, Samuels MA, Ropper AH. Adams and Victor’s principles of neurology [Internet]. McGraw-Hill Medical; 2009 [cited 2018 Feb 22]. 1572 p. Available from: https://books.google.com/books/about/Adams_and_Victor_s_Principles_of_Neurolo.html?id=AKuJoETp2_UC

[3] Pearson OR, Busse ME, van Deursen RWM, Wiles CM. Quantification of walking mobility in neurological disorders. QJM [Internet]. 2004 Aug 1 [cited 2017 Dec 18];97(8):463–475. Available from: https://academic.oup.com/qjmed/article-lookup/doi/10.1093/qjmed/hch084 10.1093/qjmed/hch084 15256604

[4] Wada O, Nagai K, Hiyama Y, Nitta S, Maruno H, Mizuno K. Diabetes is a Risk Factor for Restricted Range of Motion and Poor Clinical Outcome After Total Knee Arthroplasty. J Arthroplasty [Internet]. 2016 Sep 1 [cited 2018 Aug 9];31(9):1933–1937. Available from: https://www.sciencedirect.com/science/article/pii/S0883540316001820 10.1016/j.arth.2016.02.039 27036923

[5] Węgrzynowska-Teodorczyk Kinga, Siennicka Agnieszka, Josiak Krystian, Zymliński Robert, Kasztura Monika, Banasiak Waldemar, Ponikowski Piotr, Woźniewski Marek (2018). Evaluation of Skeletal Muscle Function and Effects of Early Rehabilitation during Acute Heart Failure: Rationale and Study Design. BioMed Research International.

[6] Du Huiyun, Newton Phillip J, Budhathoki Chakra, Everett Bronwyn, Salamonson Yenna, Macdonald Peter S, Davidson Patricia M (2017). The Home-Heart-Walk study, a self-administered walk test on perceived physical functioning, and self-care behaviour in people with stable chronic heart failure: A randomized controlled trial. European Journal of Cardiovascular Nursing.

[7] Miyamoto Seiko, Minakata Yoshiaki, Azuma Yuichiro, Kawabe Kazumi, Ono Hideya, Yanagimoto Ryuta, Suruda Tadatoshi (2018). Verification of a Motion Sensor for Evaluating Physical Activity in COPD Patients. Canadian Respiratory Journal.

[8] Ku LC, Ramli M, Abidin AMZ, Zulkifli AAN, Manaf NI, Roshini NAM, et al. Development of portable elbow joint device for stroke patient rehabilitation Physical Therapy and Rehabilitation. 2018 [cited 2018 Aug 9];5(5). Available from: http://www.hoajonline.com/journals/pdf/2055-2386-5-5.pdf

[9] Sagar VA, Davies EJ, Briscoe S, Coats AJS, Dalal HM, Lough F, et al. Exercise-based rehabilitation for heart failure: systematic review and meta-analysis. Open Hear [Internet]. 2015 Jan 1 [cited 2018 Aug 9];2(1):e000163. Available from: http://openheart.bmj.com/lookup/doi/10.1136/openhrt-2014-000163 10.1136/openhrt-2014-000163 PMC4316592 25685361

[10] Finn JM. Classical mechanics [Internet]. Jones and Bartlett Publishers; 2010 [cited 2018 Feb 22]. 576 p. Available from: http://www.jblearning.com/catalog/9780763779603/

[11] Jovanov E, Milenkovic A, Otto C, de Groen PC. A wireless body area network of intelligent motion sensors for computer assisted physical rehabilitation. J Neuroeng Rehabil [Internet]. 2005 Mar 1 [cited 2017 Dec 18];2(1):6. Available from: http://jneuroengrehab.biomedcentral.com/articles/10.1186/1743-0003-2-6 10.1186/1743-0003-2-6 PMC552302 15740621

[12] Zhou H, Hu H. Human motion tracking for rehabilitation—A survey. Biomed Signal Process Control [Internet]. 2008 Jan 1 [cited 2017 Dec 18];3(1):1–18. Available from: http://www.sciencedirect.com/science/article/pii/S1746809407000778

[13] Mantyjarvi J, Himberg J, Seppanen T. Recognizing human motion with multiple acceleration sensors. In: 2001 IEEE International Conference on Systems, Man and Cybernetics e-Systems and e-Man for Cybernetics in Cyberspace (CatNo01CH37236) [Internet]. IEEE; [cited 2018 Feb 22]. p. 747–52. Available from: http://ieeexplore.ieee.org/document/973004/

[14] Urtasun R, Fua P. 3D tracking for gait characterization and recognition. In: Sixth IEEE International Conference on Automatic Face and Gesture Recognition, 2004 Proceedings [Internet]. IEEE; [cited 2018 Feb 22]. p. 17–22. Available from: http://ieeexplore.ieee.org/document/1301503/

[15] Ferreira JP, Crisostomo MM, Coimbra AP. Human Gait Acquisition and Characterization. IEEE Trans Instrum Meas [Internet]. 2009 Sep [cited 2018 Feb 22];58(9):2979–88. Available from: http://ieeexplore.ieee.org/document/4957058/

[16] Zhu R, Zhou Z. A Real-Time Articulated Human Motion Tracking Using Tri-Axis Inertial/Magnetic Sensors Package. IEEE Trans Neural Syst Rehabil Eng [Internet]. 2004 Jun [cited 2018 Feb 22];12(2):295–302. Available from: http://ieeexplore.ieee.org/document/1304870/10.1109/TNSRE.2004.827825 15218943

[17] Cai Q, Aggarwal JK. Tracking human motion in structured environments using a distributed-camera system. IEEE Trans Pattern Anal Mach Intell [Internet]. 1999 [cited 2018 Feb 22];21(11):1241–1247. Available from: http://ieeexplore.ieee.org/document/809119/

[18] Zhang L, Sturm J, Cremers D, Lee D. Real-time human motion tracking using multiple depth cameras. In: 2012 IEEE/RSJ International Conference on Intelligent Robots and Systems [Internet]. IEEE; 2012 [cited 2018 Feb 22]. p. 2389–95. Available from: http://ieeexplore.ieee.org/document/6385968/

[19] Hale LA, Pal J, Becker I. Measuring Free-Living Physical Activity in Adults With and Without Neurologic Dysfunction With a Triaxial Accelerometer. Arch Phys Med Rehabil [Internet]. 2008 Sep 1 [cited 2017 Dec 18];89(9):1765–1771. Available from: http://www.sciencedirect.com/science/article/pii/S0003999308004292?via%3Dihub 10.1016/j.apmr.2008.02.027 18760161

[20] van der Ploeg HP, Streppel KRM, van der Beek AJ, van der Woude LH, Vollenbroek-Hutten M, van Mechelen W. The Physical Activity Scale for Individuals with Physical Disabilities: Test-Retest Reliability and Comparison with an Accelerometer. J Phys Act Heal [Internet]. 2007 Jan [cited 2017 Dec 18];4(1):96–100. Available from: http://journals.humankinetics.com/doi/10.1123/jpah.4.1.96 10.1123/jpah.4.1.96 17489011

[21] Mancini M., Zampieri C., Carlson-Kuhta P., Chiari L., Horak F. B. (2009). Anticipatory postural adjustments prior to step initiation are hypometric in untreated Parkinson’s disease: an accelerometer-based approach. European Journal of Neurology.

[22] Kim D-H, Rogers JA. Stretchable Electronics: Materials Strategies and Devices. Adv Mater [Internet]. 2008 Dec 17 [cited 2018 Feb 22];20(24):4887–4892. Available from: http://doi.wiley.com/10.1002/adma.200801788

[23] Kim D-H, Ghaffari R, Lu N, Rogers JA (2012). Flexible and stretchable electronics for biointegrated devices. Annu Rev Biomed Eng [Internet].

[24] Liu Yuhao, Norton James J. S., Qazi Raza, Zou Zhanan, Ammann Kaitlyn R., Liu Hank, Yan Lingqing, Tran Phat L., Jang Kyung-In, Lee Jung Woo, Zhang Douglas, Kilian Kristopher A., Jung Sung Hee, Bretl Timothy, Xiao Jianliang, Slepian Marvin J., Huang Yonggang, Jeong Jae-Woong, Rogers John A. (2016). Epidermal mechano-acoustic sensing electronics for cardiovascular diagnostics and human-machine interfaces. Science Advances.

[25] Kim H-J, Sim K, Thukral A, Yu C. Rubbery electronics and sensors from intrinsically stretchable elastomeric composites of semiconductors and conductors. Sci Adv [Internet]. 2017 Sep 8 [cited 2018 Feb 22];3(9):e1701114. Available from: http://advances.sciencemag.org/lookup/doi/10.1126/sciadv.1701114 10.1126/sciadv.1701114 PMC5590788 28913428

[26] Chen Y, Lu B, Chen Y, Feng X. Breathable and Stretchable Temperature Sensors Inspired by Skin. Sci Rep [Internet]. 2015 Sep 22 [cited 2018 Feb 22];5(1):11505. Available from: http://www.nature.com/articles/srep11505 10.1038/srep11505 PMC4476093 26095941

[27] Sakaguchi T, Kanamori T, Katayose H, Sato K, Inokuchi S. Human motion capture by integrating gyroscopes and accelerometers. In: 1996 IEEE/SICE/RSJ International Conference on Multisensor Fusion and Integration for Intelligent Systems (Cat No96TH8242) [Internet]. IEEE; [cited 2018 Jan 15]. p. 470–5. Available from: http://ieeexplore.ieee.org/document/572219/

[28] Godwin A, Agnew M, Stevenson J. Accuracy of Inertial Motion Sensors in Static, Quasistatic, and Complex Dynamic Motion. J Biomech Eng [Internet]. 2009 Nov 1 [cited 2018 Jan 15];131(11):114501. Available from: http://biomechanical.asmedigitalcollection.asme.org/article.aspx?articleid=1475802 10.1115/1.4000109 20353265

[29] Chen KY, Bassett DR. The Technology of Accelerometry-Based Activity Monitors: Current and Future. Med Sci Sport Exerc [Internet]. 2005 [cited 2018 Jan 15];37(11):490–500. Available from: http://citeseerx.ist.psu.edu/viewdoc/download?doi=10.1.1.462.3135&rep=rep1&type=pdf 10.1249/01.mss.0000185571.49104.82 16294112

[30] Willemsen A.T.M., Frigo C., Boom H.B.K. (1991). Lower extremity angle measurement with accelerometers-error and sensitivity analysis. IEEE Transactions on Biomedical Engineering.

[31] Cai Q, Aggarwal JK. Tracking human motion using multiple cameras. In: Proceedings of 13th International Conference on Pattern Recognition [Internet]. IEEE; 1996 [cited 2018 Jan 15]. p. 68–72 vol.3. Available from: http://ieeexplore.ieee.org/document/546796/

[32] Farouk El-Zayat B, Efe T, Heidrich A, Wolf U, Timmesfeld N, Heyse TJ, et al. Objective Assessment of shoulder mobility with a new 3D gyroscope -a validation study. BMC Musculoskelet Disord [Internet]. 2011 [cited 2016 Oct 7];12. Available from: http://www.biomedcentral.com/1471-2474/12/10.1186/1471-2474-12-168 PMC3151225 21777447

[33] SOUCIE JM, WANG C, FORSYTH A, FUNK S, DENNY M, ROACH KE, et al. Range of motion measurements: reference values and a database for comparison studies. Haemophilia [Internet]. 2011 May 1 [cited 2018 Jan 15];17(3):500–507. Available from: http://doi.wiley.com/10.1111/j.1365-2516.2010.02399.x 10.1111/j.1365-2516.2010.02399.x 21070485

[34] Boone DC, Azen SP. Normal range of motion of joints in male subjects. J Bone Joint Surg Am [Internet]. 1979 [cited 2016 Sep 29];61(5):756–759. Available from: http://www.ncbi.nlm.nih.gov/entrez/query.fcgi?cmd=Retrieve&db=PubMed&dopt=Citation&list_uids=457719 457719

[35] Gajdosik RL, Bohannon RW. Clinical Measurement of Range of Motion. Phys Ther [Internet]. 1987 Dec 1 [cited 2018 Jan 15];67(12):1867–1872. Available from: https://academic.oup.com/ptj/article-lookup/doi/10.1093/ptj/67.12.1867 10.1093/ptj/67.12.1867 3685114

[36] Greene B, Mckeon CLTK. A THREE-DIMENSIONAL KINEMATIC COMPARISON OF PITCHING TECHNIQUES BETWEEN MALE AND FEMALE FAST-PITCH SOFTBALL . PLAYERS. [cited 2018 Apr 2]; Available from: https://ojs.ub.uni-konstanz.de/cpa/article/viewFile/2557/2406

[37] Adams Paul S., Keyserling W.Monroe (1993). Three methods for measuring range of motion while wearing protective clothing: A comparative study. International Journal of Industrial Ergonomics.

[38] El-Zayat BF, Efe T, Heidrich A, Wolf U, Timmesfeld N, Heyse TJ, et al. Objective Assessment of shoulder mobility with a new 3D gyroscope - a validation study. BMC Musculoskelet Disord [Internet]. 2011 Dec 21 [cited 2018 Jan 15];12(1):168. Available from: http://bmcmusculoskeletdisord.biomedcentral.com/articles/10.1186/1471-2474-12-168 10.1186/1471-2474-12-168 PMC3151225 21777447

[39] Armstrong AD, MacDermid JC, Chinchalkar S, Stevens RS, King GJW (1998). Reliability of range-of-motion measurement in the elbow and forearm. J Shoulder Elb Surg.

[40] Stillman BC, Tully EA, McMeeken JM. Knee Joint Mobility and Position Sense in Healthy Young Adults. Physiother Sept [Internet]. 2002 [cited 2018 Mar 27];88(9). Available from: http://www.physiotherapyjournal.com/article/S0031-9406(05)60138-1/pdf

[41] Levine MG, Kabat H. Proprioceptive Facilitation of Voluntary Motion in Man [Internet]. Williams & Wilkins; [cited 2018 Mar 27]. Available from: http://zp9vv3zm2k.scholar.serialssolutions.com/?sid=google&auinit=MG&aulast=Levine&atitle=Proprioceptive+facilitation+of+voluntary+motion+in+man.&title=The+journal+of+nervous+and+mental+disease&volume=117&issue=3&date=1953&spage=199&issn=0022-3018 10.1097/00005053-195303000-00002 13070034

[42] Garlant JA, Ammann KR, Slepian MJ. Stretchable Electronic Wearable Motion Sensors Delineate Signatures of Human Motion Tasks. ASAIO J [Internet]. 2018 [cited 2019 Aug 7];64(3):351–359. Available from: http://insights.ovid.com/crossref?an=00002480-201805000-00012 10.1097/MAT.0000000000000784 PMC6128668 29608494

[43] Nikravesh PE, Wehage RA, Kwon OK (1985). Euler parameters in computational kinematics and dynamics. Part 1. J Mech Transm Autom Des.

