# Supplementary material for: Human motion component and envelope characterization via wireless wearable sensors
Source: BMC Biomed Eng. 2020 Feb 27;2:3. doi: 10.1186/s42490-020-0038-4 (PMC7422588; doi:10.1186/s42490-020-0038-4)
Supplement: Supplementary file 1 — Additional file 1: Table S1. Wearable Motion Capture Sensor Specifications. Table S2. Wearable Fitness Tracker Capabilities. Figure S1. 2-D Trajectories of Human Arm Motion. Figure S2. Motion Signatures of Gross Human Activity Series. Flow Diagram of Motion Data Processing. [file 42490_2020_38_MOESM1_ESM.pdf]

Table S1. Wearable Motion Capture Sensor Specifications

Sensor	Producing Company	Measurement Capability	Sampling Rate	Battery Life (hrs)	Weight (g)	Dimensions (L x W x D cm)	Memory	Range	Resolution
**BioStampRC**	MC10	Accelerometer<br>Gyroscope<br>ECG<br>EMG	**Accelerometer/ Gyroscope**<br>15.625 - 250 Hz<br>**ECG/EMG**<br>125 - 1000 Hz	36	7	6.6 x 3.4 x 0.45	32 MB (Flash)	**Accelerometer**<br>± 16 g<br>**Gyroscope**<br>±2000 °/s<br>**ECG/EMG**<br>± 200 mV	**Accelerometer**<br>5 mg<br>**Gyroscope**<br>0.2 °/s<br>**ECG/EMG**<br>0.006 mV
**MotionNode Bus**	MotionNode	Accelerometer<br>Gyroscope<br>Magnetometer	**Accelerometer/ Gyroscope**<br>50 Hz	7	190	3.5 x 3.5 x 1.5	4 GB	**Accelerometer**<br>± 2 g or ± 6 g<br>**Gyroscope**<br>±2000 °/s<br>**Magnetometer**<br>± 100 µT	**Accelerometer**<br>190 µg ± 5%<br>**Gyroscope**<br>0.07 °/s<br>**Magnetometer**<br>0.1 µT
**Colibri Wireless**	Engineering Systems Technologie s	Accelerometer<br>Gyroscope<br>Magnetometer<br>Static Pressure<br>Temperature	**Accelerometer/ Gyroscope**<br>100 Hz	16	41	5.6 x 4.2 x 1.7	1 KB	**Accelerometer**<br>± 6 g<br>**Gyroscope**<br>± 2000 °/s<br>**Magnetometer**<br>± 1.3 Ga	**Accelerometer**<br>13 bit<br>**Gyroscope**<br>16 bit<br>**Magnetometer**<br>12 bit
**Opal**	APDM Wearable Technologie s	Accelerometer<br>Gyroscope<br>Magnetometer	**Accelerometer/ Gyroscope**<br>20 - 128 Hz	12	25	4.8 x 4.0 x 1.4	1 GB	**Accelerometer**<br>±16g, ± 200g<br>**Gyroscope**<br>± 2000 °/s<br>**Magnetometer**<br>± 8 Ga	**Accelerometer**<br>14 bit, 17.5 bit<br>**Gyroscope**<br>16 bit<br>**Magnetometer**<br>12 bit
**Embrace2**	Empatica	Accelerometer<br>Gyroscope<br>Temperature	**Accelerometer/ Gyroscope**<br>32 Hz	48	17	3.8 x 3.0 x 1.0	1 MB	**Accelerometer**<br>±16g<br>**Gyroscope**<br>± 500 °/s	**Accelerometer**<br>16 bits<br>**Gyroscope**<br>16 bits
**Shimmer3 IMU Unit**	Shimmer	Accelerometer<br>Gyroscope<br>Magnetometer<br>Altimeter<br>Temperature	**Accelerometer/ Gyroscope**<br>10 - 1000 Hz<br>**Magnetometer**<br>250 -1000 Hz	70	24	5.1 x 3.4 x 1.4	16 KB	**Accelerometer**<br>± 2 - 16 g<br>**Gyroscope**<br>± 250 - 2000 °/s<br>**Magnetometer**<br>± 49 Ga	**Accelerometer**<br>14 bits<br>**Gyroscope**<br>16 bits<br>**Magnetometer**<br>16 bits

**BioHarness 3.0**	Zephyr	Accelerometer<br>ECG<br>Temperature	**Accelerometer**<br>100 Hz<br>**ECG**<br>250Hz	24	89	2.8 x 2.8 x 0.7	--	**Accelerometer**<br>±16g<br>**ECG**<br>0.25 – 15 mV	**Accelerometer**<br>±12mg<br>**ECG**<br>12 bit
**Physilog 5**	GaitUp	Accelerometer<br>Gyroscope<br>Barometer<br>Temperature	**Accelerometer/ Gyroscope**<br>128 Hz	21	11	4.8 x 2.7 x 1.0	8 GB	**Accelerometer**<br>± 16 g<br>**Gyroscope**<br>±2000 °/s	--
**EQ02 LifeMonitor**	Equivital	Accelerometer<br>ECG<br>Temperature	**Accelerometer**<br>25 – 250 Hz<br>**ECG**<br>250Hz	24	38	7.8 x 5.3 x 1.0	8 GB	**Accelerometer**<br>±2g - 16g	**--**
**MTw Awinda**	Xsens	Accelerometer<br>Gyroscope<br>Magnetometer<br>Barometer	**Accelerometer/ Gyroscope**<br>1000 Hz	6	27	3.0 x 4.7 x 1.3	--	**Accelerometer**<br>± 16 g<br>**Gyroscope**<br>± 2000 °/s<br>**Magnetometer**<br>± 1.9 Ga	**--**

ECG = Electrocardiogram; EMG = electromyogram

Table S2. Wearable Fitness Tracker Capabilities

Sensor	Producing Company	Measurement Capability	Weight (g)	Dimensions (L x W x D cm)	Memory	Data Output	Motion Capabilities
**BioStampRC**	MC10	Accelerometer<br>Gyroscope<br>ECG<br>EMG	7	6.6 x 3.4 x 0.45	32 MB (Flash)	Raw data	6 DOF
**Charge 3**	Fitbit	Accelerometer<br>Gyroscope<br>Altimeter<br>PPG HR	30	3.8 x 1.8 x 1.2	2 GB	Activity<br>Calories burned<br>Step count<br>Distance<br>Elevation change<br>Heart rate	6 DOF
**Apple Watch Series 5**	Apple	Accelerometer<br>Gyroscope<br>Barometer<br>ECG<br>PPG HR	37	4.4 x 3.8 x 1.1	32 GB	Activity<br>Calories burned<br>Step count<br>Distance<br>Elevation change<br>Filtered ECG<br>Heart rate variability<br>Heart rate	6 DOF
**Vivosmart HR**	Garmin	Accelerometer<br>Barometer<br>PPG HR	32	13.6 x 19.2	3 GB	Activity<br>Calories burned<br>Step count<br>Distance<br>Elevation change<br>Heart rate	3 DOF

ECG = Electrocardiogram; EMG = electromyogram; PPG = photoplethysmography; HR = heart rate; DOF = degrees of freedom

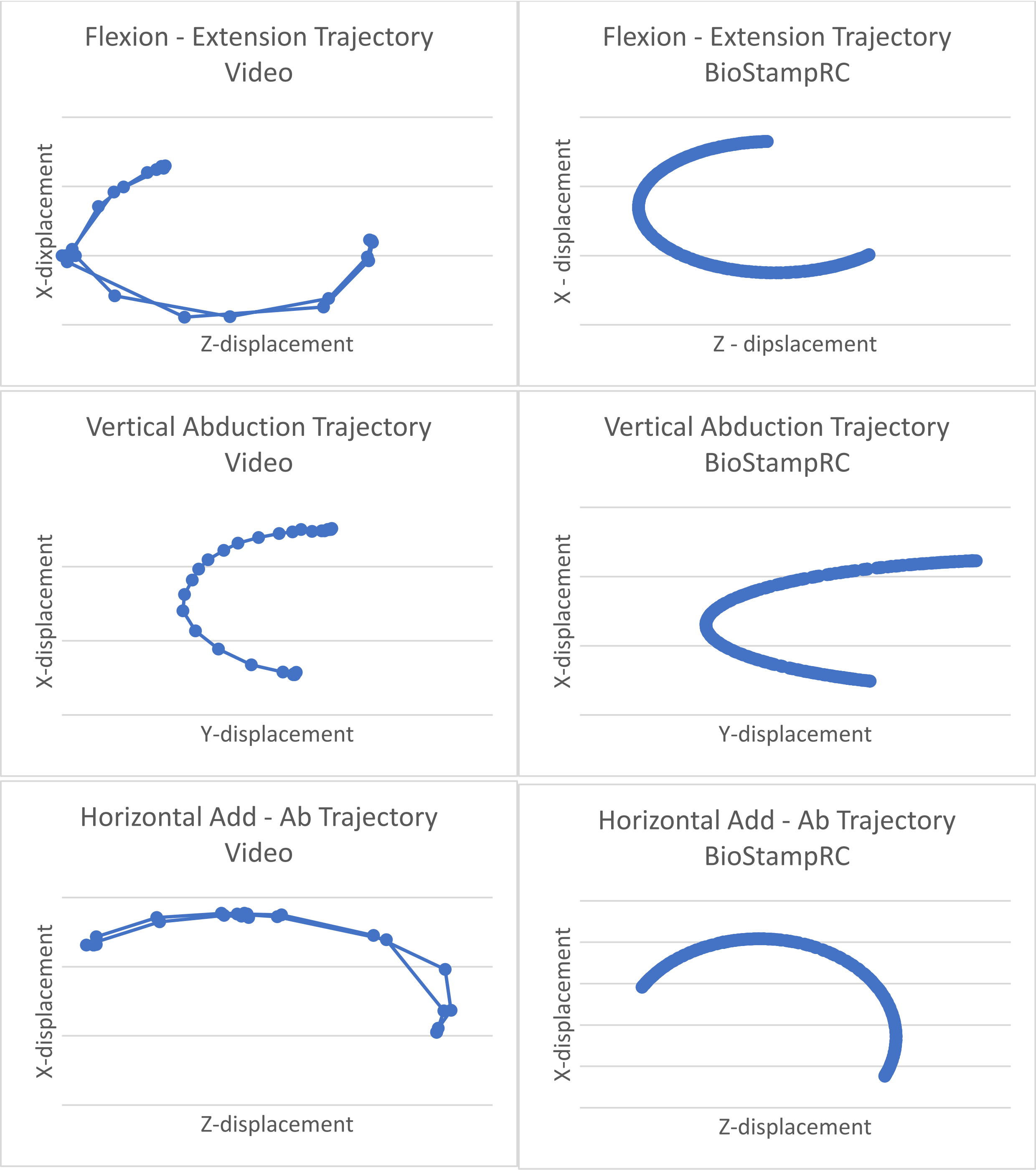

Fig S1. 2-D Trajectories of Human Arm Motion. (Left column) 2-dimensional trajectory of arm from video motion tracking compared to (right column) trajectory calculated from BioStampRC gyroscope data.

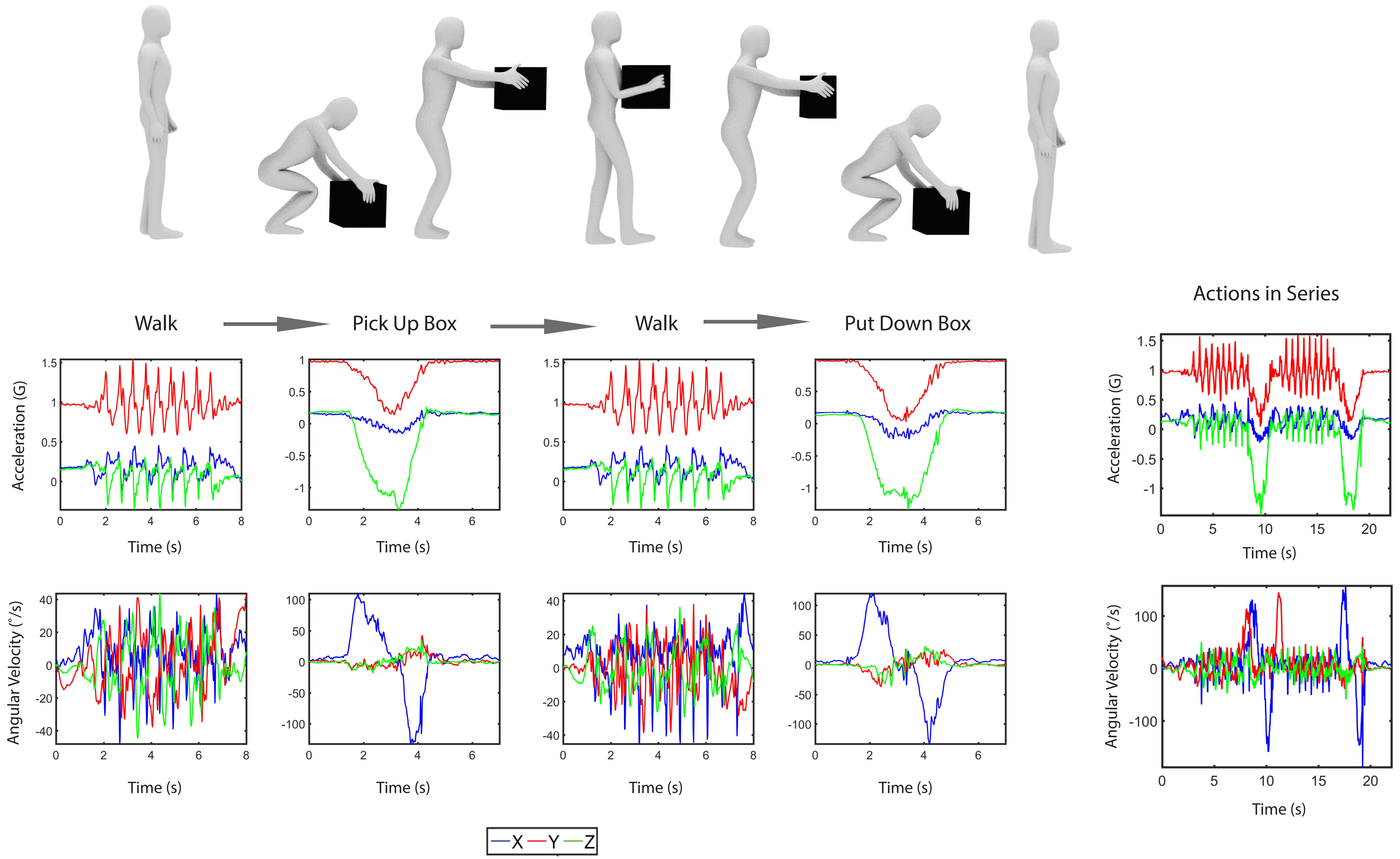

Fig S2. Motion Signatures of Gross Human Activity Series. (Top row) Tri-axial acceleration motion signature and (bottom row) tri-axial angular velocity motion signature of human subject performing basic motion activity separately and in series.

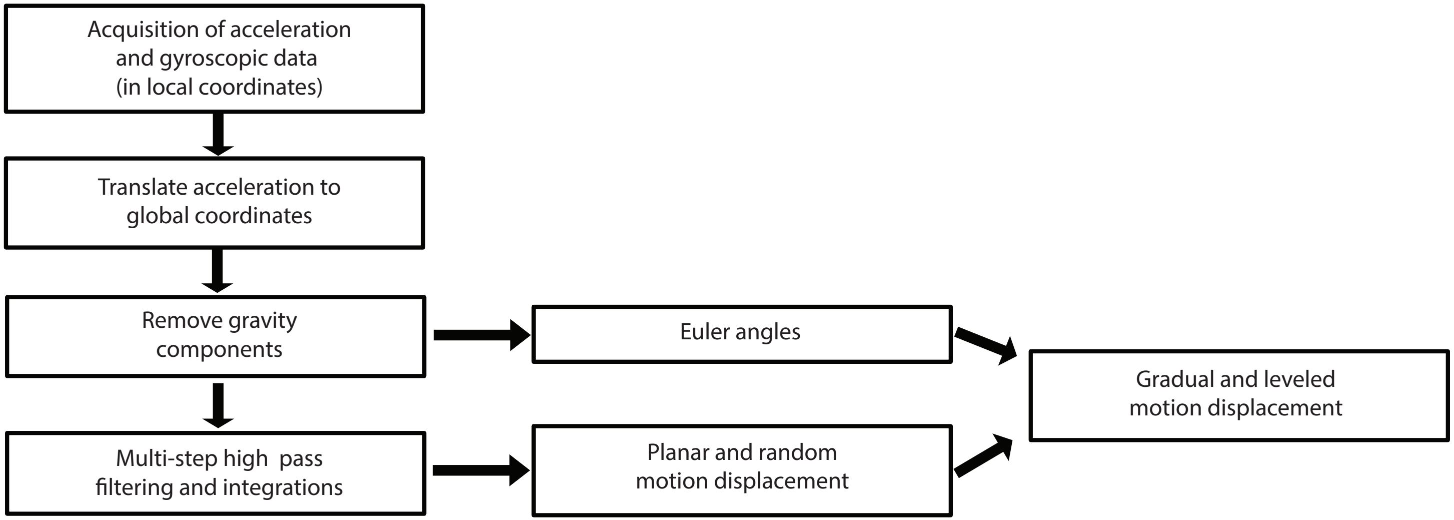

Fig S3. Flow Diagram of Motion Data Processing.
